# Supplementary material for: The CBL1/9-CIPK1 calcium sensor negatively regulates drought stress by phosphorylating the PYLs ABA receptor
Source: Nat Commun. 2023 Sep 21;14:5886. doi: 10.1038/s41467-023-41657-0 (PMC10514306; doi:10.1038/s41467-023-41657-0)
Supplement: Supplementary file 1 — Supplementary Information [file 41467_2023_41657_MOESM1_ESM.pdf]

The CBL1/9-CIPK1 calcium sensor negatively regulates drought stress by phosphorylating the PYLs ABA receptor

Zhang You[1], Shiyuan Guo[1], Qiao Li[1], Yanjun Fang[1], Panpan Huang[1], Chuanfeng Ju[1] and Cun Wang[1, 2]*.

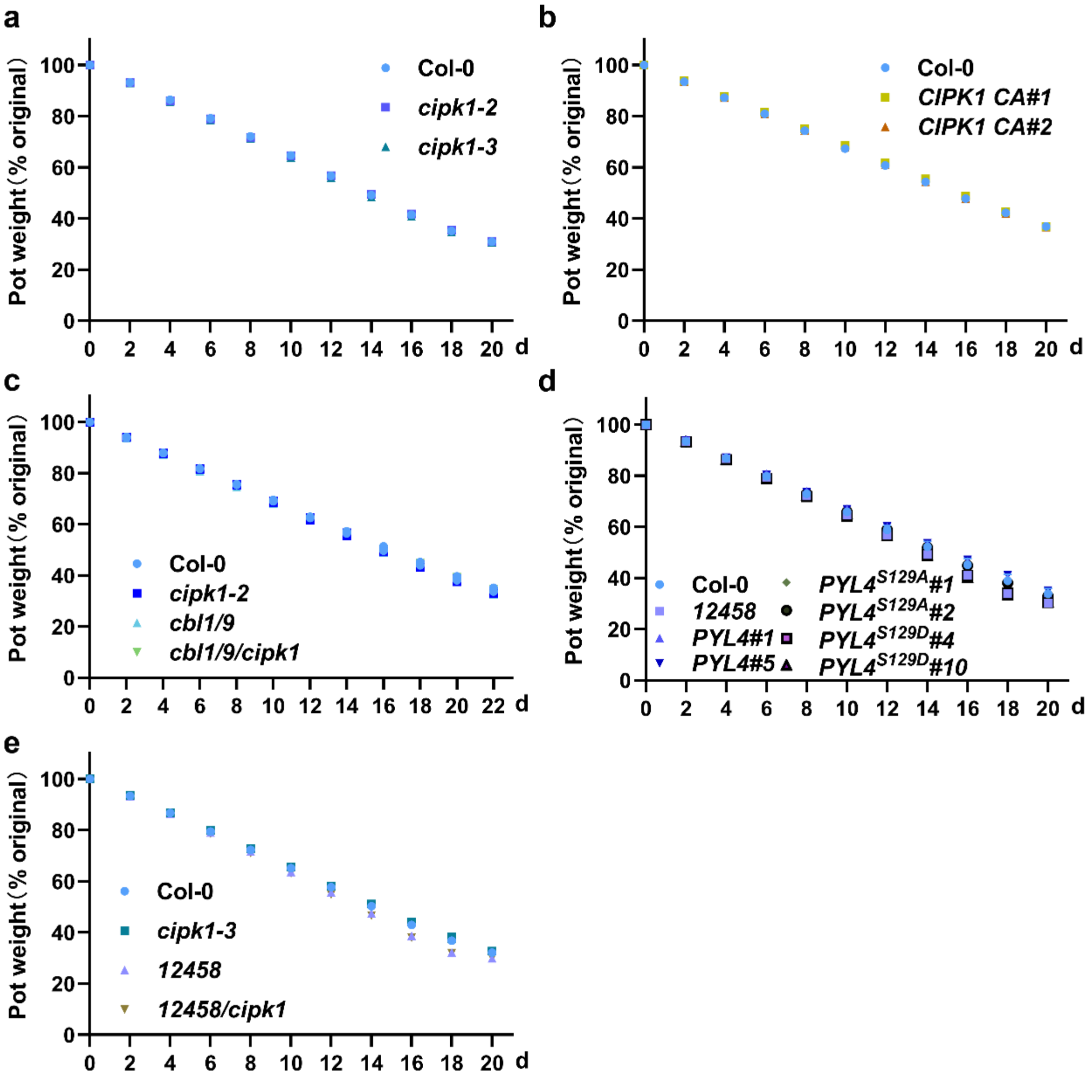

Supplementary Fig.1. Measurement of soil water content during drought treatments.

(a-e) Pot weight for drought assays was measured and plotted as a percentage of the original weight. The experiment was repeated three times with independent treatments. Data are expressed as mean ± SD.

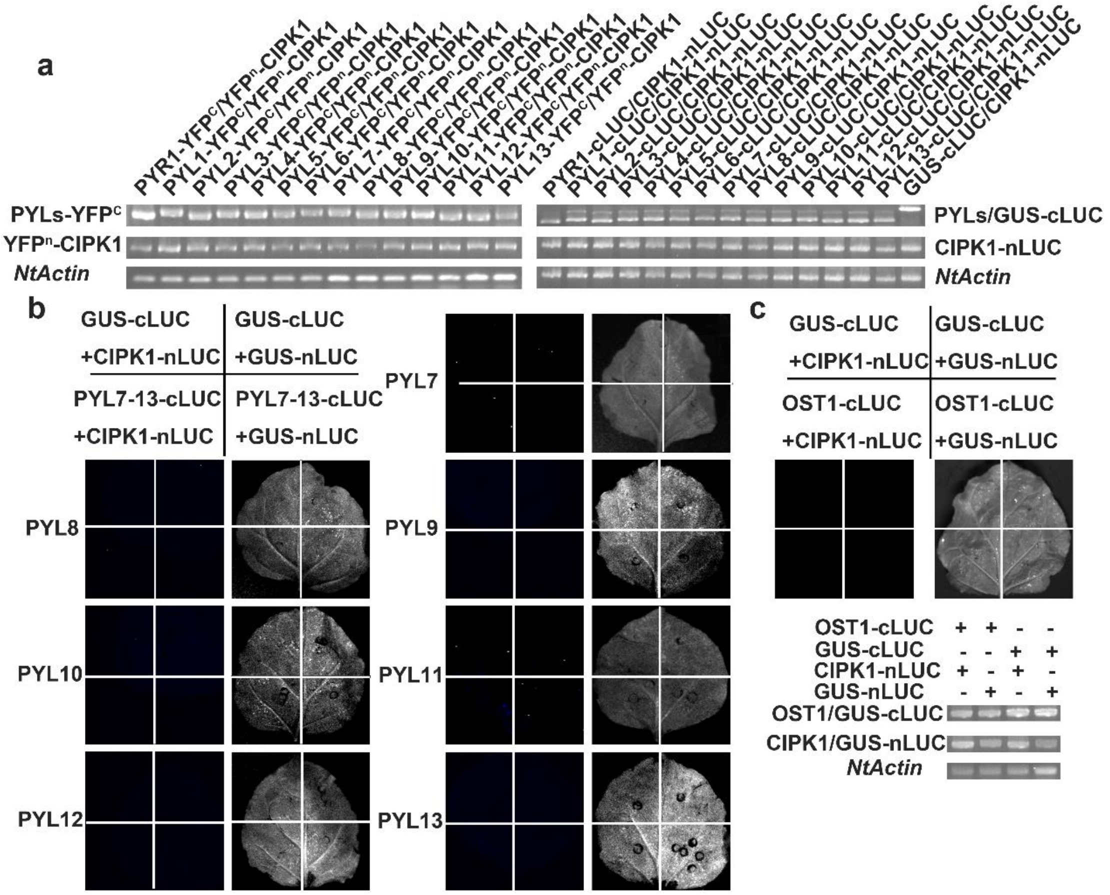

Supplementary Fig. 2. CIPK1 does not interact with PYL7-13 and OST1.

(a) Semi-qRT-PCR analysis of CIPK1, PYLs, and GUS in the transiently infiltrated *N. benthamiana* leaves shown in (Fig. 2a, c). *NtActin* was used as a control.

(b) CIPK1 does not interact with PYL7-13 in LCI assay.

(c) OST1 does not interact with CIPK in LCI assay. The experiments were repeated three times (a–c) with similar results.

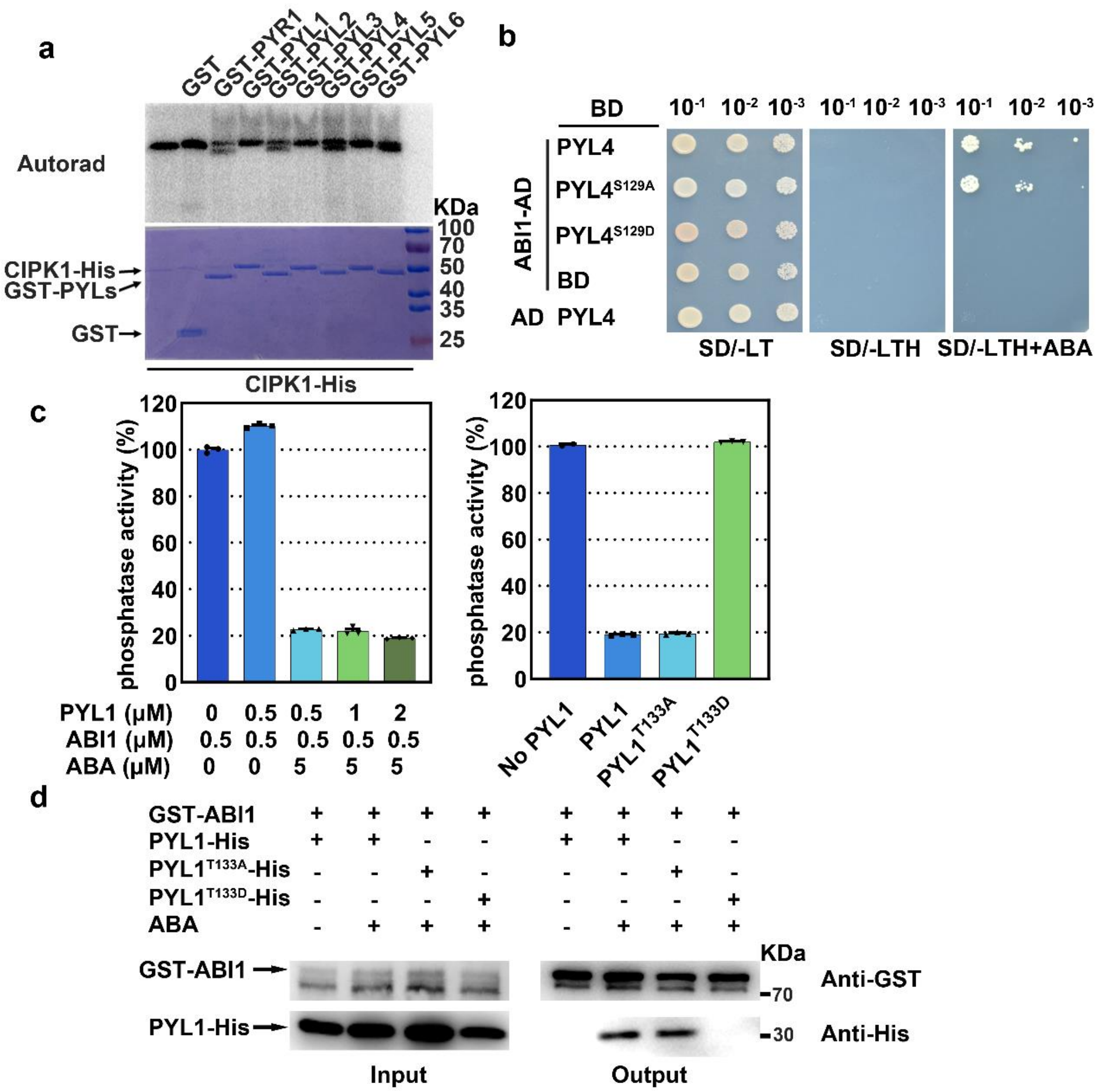

Supplementary Fig. 3. CIPK1 phosphorylates PYLs inhibiting their activity *in vitro*. Attached to Fig. 3.

(a) CIPK1 phosphorylation of PYR1 and PYL2 *in vitro*. In the assay, 1 μg CIPK1-His and 1.5 μg GST-PYLs were incubated for 30 min at 30°C in a kinase buffer supplemented with 2 μCi [γ-^{32}P] ATP. The proteins were separated by 12% SDS-PAGE. The experiment was repeated two times with similar results

(b) PYL4 and PYL4^{S129A} but not PYL4^{S129D} interacted with ABI1 in yeast two-hybrid assay. The experiment was repeated thrice with similar results.

(c) PYL1^{T133D} did not inhibit the phosphatase activity of ABI1. Data are shown as mean ± SE. Three independent replications of the experiment obtained similar results.

(d) PYL1^{T133D} did not interact with ABI1 in pull-down assays. The experiment was performed three times with similar results.

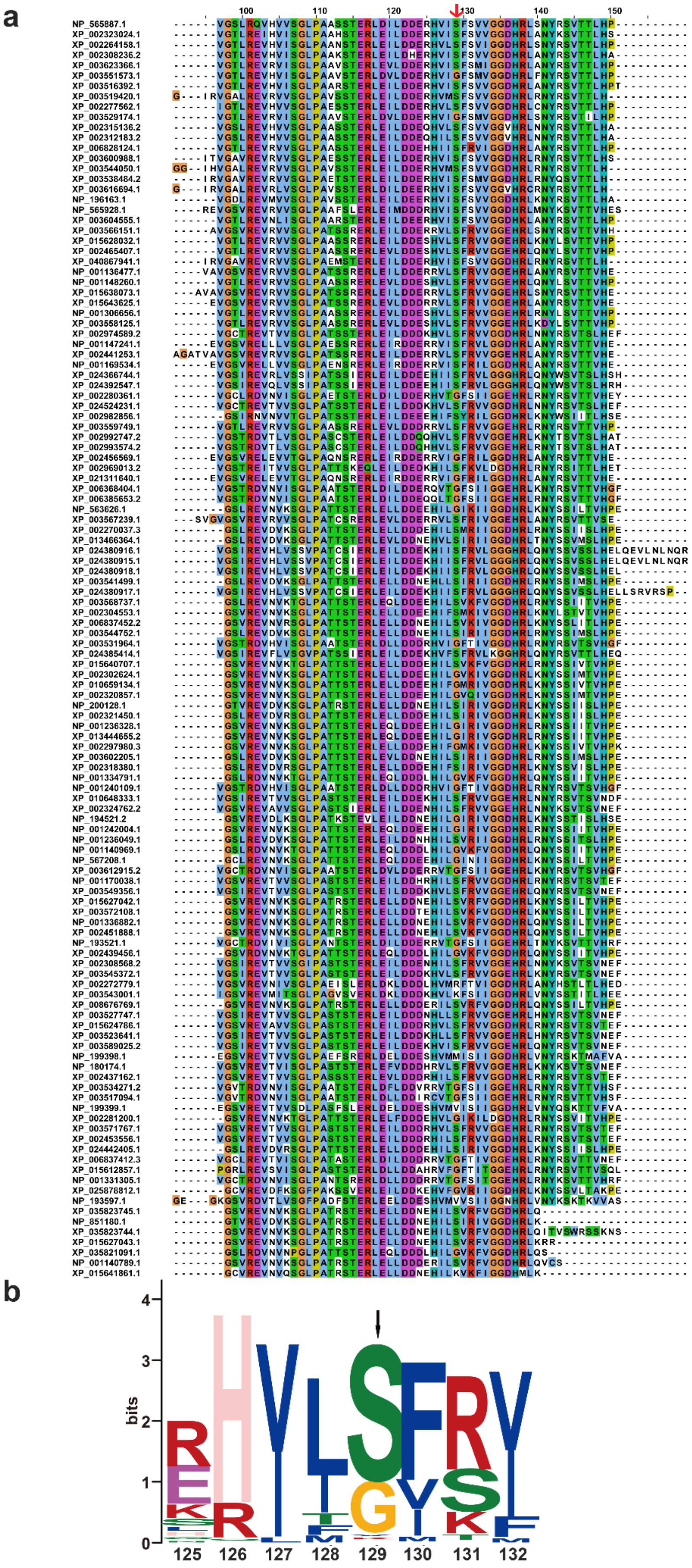

Supplementary Fig. 4. The serine corresponding to Ser129 in PYL4 is

evolutionarily conserved.

(a) Serine corresponding to Ser129 in PYL4 is evolutionarily conserved in 122 PYLs in 12 different species including *Arabidopsis thaliana*, *Vitis vinofera*, *Medicago truncatula*, *Glycine max*, *Populus trichocarpa, Oryza sativa*, *Zea mays*, *Sorghum bicolor*, *Brachypodium distachyon*, *Amborella trichopoda, Selaginella moellendorffii*, *and Physcomitrella patens*.

(b) Conservative analysis of PYL4 Ser129.

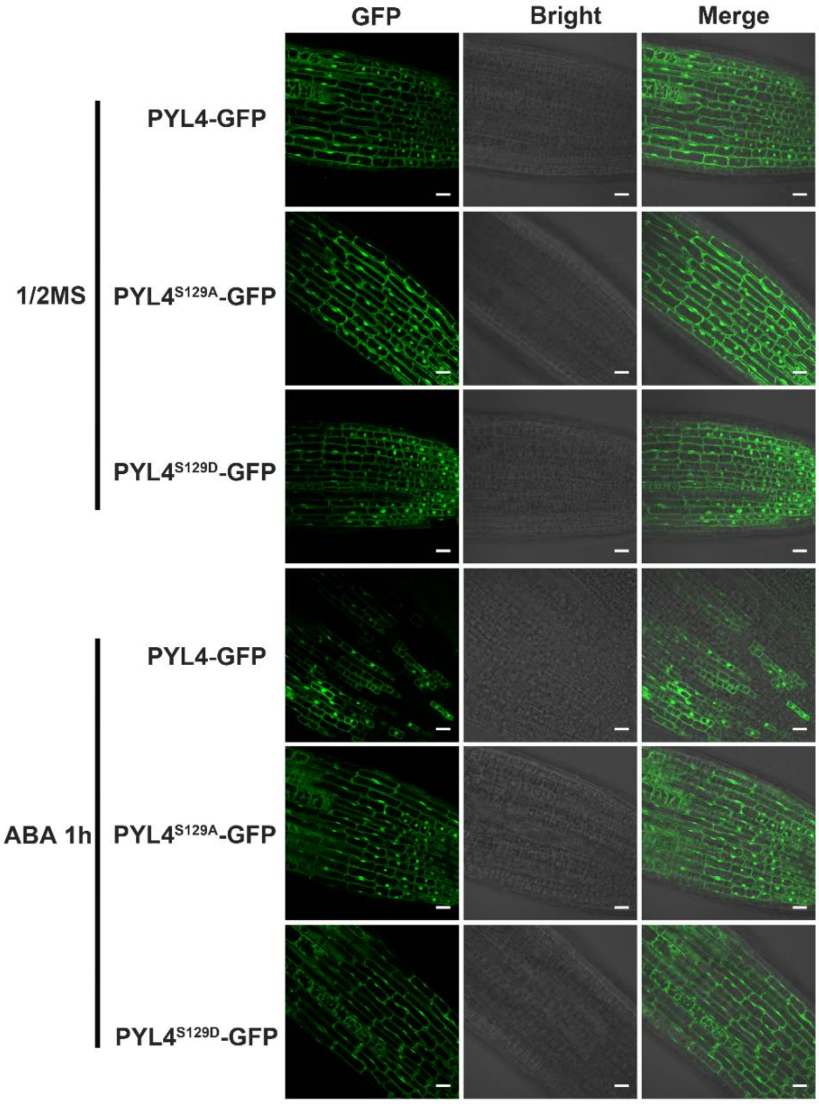

Supplementary Fig. 5. CIPK1 does not regulate the localization of PYL4. Confocal microscopy analysis of the localization of PYL4, PYL4^{S129A}, and PYL4^{S129D} with or without 60 μM ABA treatment. Scale bars, 40 μm. The experiment was repeated three times and similar results were obtained.

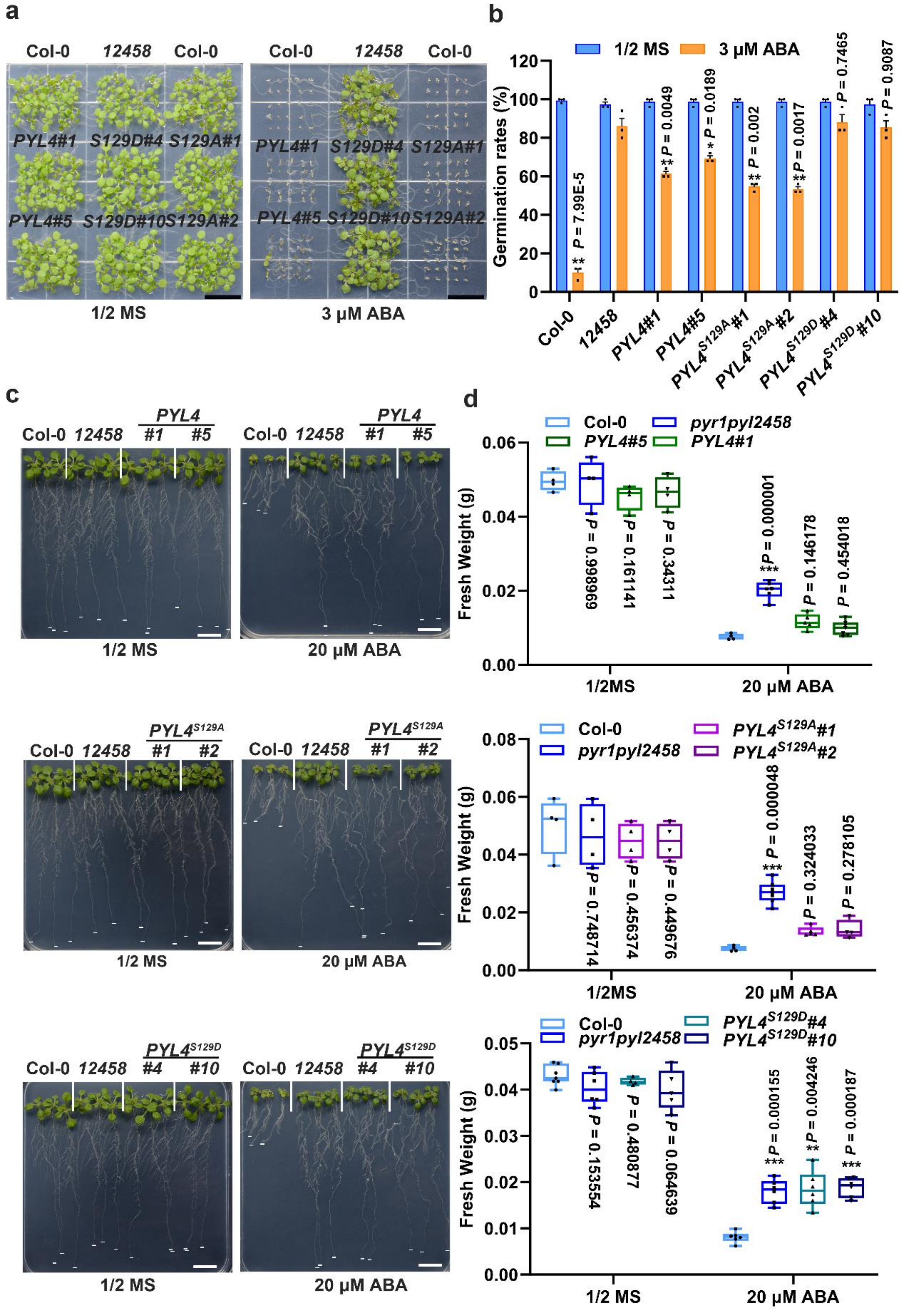

Supplementary Fig. 6. Ser129 was crucial in response to ABA.

(a) 10-day-old seedlings grown on 1/2 Murashige-Skoog (MS) medium with or without 3 μM ABA. Scale bar, 1.4 cm. The experiment was repeated thrice with similar results.

(b) Germination rate of seeds treated as in (a). Radicle emergence rate was assessed on the 3rd day after placing. Data are means ± SEM of three biological replicates. 25 seeds were analyzed in each replicate. Student's t-test (Two-sided, ** *P* <0.01), compared with *pyr1pyl2458*(*12458*).

(c) The root elongation of PYL4 complementary transgenic plants did not significantly change in ABA treatment. 4-day-old seedlings were transferred to 1/2 MS plates with or without 20 μM ABA and grew vertically for another 10 days. The root elongations were measured using ImageJ. Scale bar, 1.4 cm. Three repetitions obtained similar results.

(d) Statistics on fresh weight of plants in (c) respectively. Box plots show the 25% quantile, median (line), and 75% quantile. The whiskers plots (Tukey method) represent minimum and maximum values. Asterisks indicate statistical significance between the samples (Two-way ANOVA analysis by Dunnett's multiple comparisons tests, ** *P* < 0.01, *** *P* < 0.001).

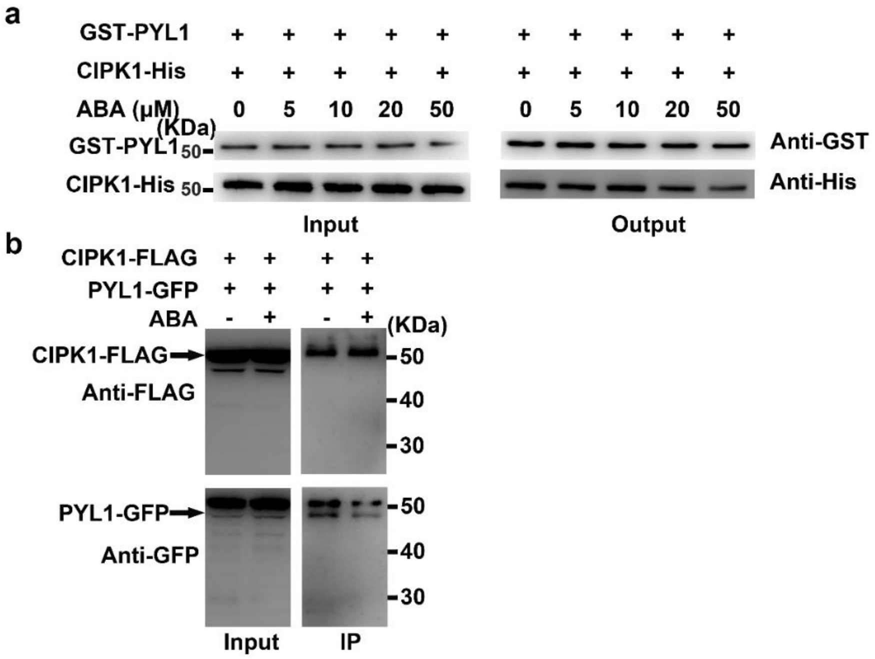

Supplementary Fig. 7. Inhibition of the interaction between CIPK1 and PYL1 by ABA.

(a) Increasing concentrations of ABA inhibits the interaction between CIPK1 and PYL1 in pull-down assays.

(b) ABA inhibits the interaction between CIPK1 and PYL1 in Co-IP assays. Each of the experiments was repeated three times with similar results.
