# Supplementary material for: The CBL1/9-CIPK1 calcium sensor negatively regulates drought stress by phosphorylating the PYLs ABA receptor
Source: Nat Commun. 2023 Sep 21;14:5886. doi: 10.1038/s41467-023-41657-0 (PMC10514306; doi:10.1038/s41467-023-41657-0)
Supplement: Supplementary file 3 — Description of Additional Supplementary Files [file 41467_2023_41657_MOESM3_ESM.pdf]

Description of Additional Supplementary Files

Supplementary Data 1. Primers used in this study.
