# Supplementary material for: The CBL1/9-CIPK1 calcium sensor negatively regulates drought stress by phosphorylating the PYLs ABA receptor
Source: Nat Commun. 2023 Sep 21;14:5886. doi: 10.1038/s41467-023-41657-0 (PMC10514306; doi:10.1038/s41467-023-41657-0)
Supplement: Supplementary file 5 — Reporting Summary [file 41467_2023_41657_MOESM5_ESM.pdf]

nature portfolio

Corresponding author(s): Cun Wang

Last updated by author(s): Sep 6, 2023

Reporting Summary

Nature Portfolio wishes to improve the reproducibility of the work that we publish. This form provides structure for consistency and transparency in reporting. For further information on Nature Portfolio policies, see our Editorial Policies and the Editorial Policy Checklist.

Statistics

For all statistical analyses, confirm that the following items are present in the figure legend, table legend, main text, or Methods section.

n/a	Confirmed	
☐	☒	The exact sample size (*n*) for each experimental group/condition, given as a discrete number and unit of measurement
☐	☒	A statement on whether measurements were taken from distinct samples or whether the same sample was measured repeatedly
☐	☒	The statistical test(s) used AND whether they are one- or two-sided<br>*Only common tests should be described solely by name; describe more complex techniques in the Methods section.*
☒	☐	A description of all covariates tested
☐	☒	A description of any assumptions or corrections, such as tests of normality and adjustment for multiple comparisons
☐	☒	A full description of the statistical parameters including central tendency (e.g. means) or other basic estimates (e.g. regression coefficient) AND variation (e.g. standard deviation) or associated estimates of uncertainty (e.g. confidence intervals)
☐	☒	For null hypothesis testing, the test statistic (e.g. *F*, *t*, *r*) with confidence intervals, effect sizes, degrees of freedom and *P* value noted<br>*Give P values as exact values whenever suitable.*
☒	☐	For Bayesian analysis, information on the choice of priors and Markov chain Monte Carlo settings
☒	☐	For hierarchical and complex designs, identification of the appropriate level for tests and full reporting of outcomes
☒	☐	Estimates of effect sizes (e.g. Cohen's *d*, Pearson's *r*), indicating how they were calculated

Our web collection on statistics for biologists contains articles on many of the points above.

Software and code

Policy information about availability of computer code

Data collection	Image J software was used to collect stomatal aperture data .Leaf temperature was measured using FLIR ResearchIR Max professional software. Confocal images were captured by the Olympus IX83-FV3000confocal microscope. LUC signals were captured by Lumazone Pylon 2048B. The absorbance was measured using a BioTek microplate spectrophotometer. The phosphorylation signals were detected with a Typhoon 9410 imager. Protein sequences were collected in NCBI.
Data analysis	Quantification and statistical analyses were performed using Fiji (Image J), and Graph Pad Prism 9.0. Bar charts and plot boxes were generated by GraphPad Prism. Adobe Photoshop CC 2018 and Adobe Illustrator were used for image assembly. Protein sequences were aligned with Jalview software. Amino acid conservativeness analysis was performed using the MEME online tool.

For manuscripts utilizing custom algorithms or software that are central to the research but not yet described in published literature, software must be made available to editors and reviewers. We strongly encourage code deposition in a community repository (e.g. GitHub). See the Nature Portfolio guidelines for submitting code & software for further information.

Data

Policy information about availability of data

All manuscripts must include a data availability statement. This statement should provide the following information, where applicable:

- Accession codes, unique identifiers, or web links for publicly available datasets
- A description of any restrictions on data availability
- For clinical datasets or third party data, please ensure that the statement adheres to our policy

All data generated and analyzed in this study are available in the article and its supplementary information file. These data can be obtained from Source Data file. Source data are provided with this paper.

Research involving human participants, their data, or biological material

Policy information about studies with human participants or human data. See also policy information about sex, gender (identity/presentation), and sexual orientation and race, ethnicity and racism.

Reporting on sex and gender	N/A
Reporting on race, ethnicity, or other socially relevant groupings	N/A
Population characteristics	N/A
Recruitment	N/A
Ethics oversight	N/A

Note that full information on the approval of the study protocol must also be provided in the manuscript.

Field-specific reporting

Please select the one below that is the best fit for your research. If you are not sure, read the appropriate sections before making your selection.

[x] Life sciences [ ] Behavioural & social sciences [ ] Ecological, evolutionary & environmental sciences

For a reference copy of the document with all sections, see nature.com/documents/nr-reporting-summary-flat.pdf

Life sciences study design

All studies must disclose on these points even when the disclosure is negative.

Sample size	Statistical methods were used to predetermine sample sizes. The exact number of samples in each experiment was specified in the figure legends. In all cases, sample sizes were adequate as the results were reproducible between different experimental groups.
Data exclusions	No data were excluded.
Replication	Each experiment was repeated for at least two or three times with similar results obtained. Number of repeats in each experiment was provided in the figure legends.
Randomization	Plants of different genotypes were randomly positioned in the growth chamber or in the greenhouse. Randomization was not applied to western blot and kinase assays.
Blinding	Blinding was impossible because the author who conducted the experiments also performed data acquisition and analysis.

Reporting for specific materials, systems and methods

We require information from authors about some types of materials, experimental systems and methods used in many studies. Here, indicate whether each material, system or method listed is relevant to your study. If you are not sure if a list item applies to your research, read the appropriate section before selecting a response.

Materials & experimental systems

n/a	Involved in the study
☐	☒ Antibodies
☒	☐ Eukaryotic cell lines
☒	☐ Palaeontology and archaeology
☒	☐ Animals and other organisms
☒	☐ Clinical data
☒	☐ Dual use research of concern
☐	☒ Plants

Methods

n/a	Involved in the study
☒	☐ ChIP-seq
☒	☐ Flow cytometry
☒	☐ MRI-based neuroimaging

Antibodies

Antibodies used: nti-GST Mouse Monoclonal Antibody: TransGene, HT601; Anti-His Mouse Monoclonal Antibody: TransGene, HT501; Anti-FLAG Antibody: 1:5000, TransGene, HT201; Anti-GFP Mouse Monoclonal Antibody: 1:5000, TransGene, HT801; Anti-β-Tubulin Mouse Monoclonal Antibody: 1:5000, TransGene, HC101; Goat Anti-Mouse IgG (H+L), HRP Conjugate: 1:5000, TransGene, HS201; Phosphoserine Monoclonal Antibody, 1: 2000, Immunoway, YM3440.

Validation: All the antibodies are commercially available from the manufacturer.

Dual use research of concern

Policy information about dual use research of concern

Hazards

Could the accidental, deliberate or reckless misuse of agents or technologies generated in the work, or the application of information presented in the manuscript, pose a threat to:

No	Yes
☒	☐ Public health
☒	☐ National security
☒	☐ Crops and/or livestock
☒	☐ Ecosystems
☒	☐ Any other significant area

Experiments of concern

Does the work involve any of these experiments of concern:

No	Yes
☒	☐ Demonstrate how to render a vaccine ineffective
☒	☐ Confer resistance to therapeutically useful antibiotics or antiviral agents
☒	☐ Enhance the virulence of a pathogen or render a nonpathogen virulent
☒	☐ Increase transmissibility of a pathogen
☒	☐ Alter the host range of a pathogen
☒	☐ Enable evasion of diagnostic/detection modalities
☒	☐ Enable the weaponization of a biological agent or toxin
☒	☐ Any other potentially harmful combination of experiments and agents
